# Commentary: A Conserved Role for Serotonergic Neurotransmission in Mediating Social Behavior in Octopus

**DOI:** 10.3389/fnbeh.2019.00185

**Published:** 2019-08-14

**Authors:** Piero Amodio, Graziano Fiorito, Nicola S. Clayton, Ljerka Ostojić

**Affiliations:** ^1^Department of Psychology, University of Cambridge, Cambridge, United Kingdom; ^2^Department of Biology and Evolution of Marine Organisms, Stazione Zoologica Anton Dohrn, Naples, Italy; ^3^Institute for Globally Distributed Open Research and Education (IGDORE), Rijeka, Croatia

**Keywords:** octopus, 3, 4-methylendioxymethamphetamine, MDMA, prosocial effects, social behavior

In a recent study with potential wide-reaching influence, (Edsinger and Dölen, 2018) tested, for the first time, the effect of 3,4-methylendioxymethamphetamine (MDMA) in the cephalopod mollusk *Octopus bimaculoides*. In their main experiment (Experiment 2), the authors placed octopuses in the central compartment of a three-chambered arena and allowed them to freely explore the lateral chambers, one containing an object and the other containing a social stimulus (a familiar male conspecific), both isolated through a perforated plastic container. All subjects first received a pre-trial to establish a baseline for the social response toward the conspecific, and following the administration of MDMA, they were given a post-trial with the same individual. According to the authors, the results demonstrate that MDMA induces both quantitative (i.e., longer intervals spent in the social stimulus chamber) and qualitative (i.e., different behaviors) acute prosocial responses in octopus. Here we highlight fundamental flaws in the study, thus challenging the authors' conclusions.

Foremost, this experiment foregoes the standard procedure in establishing causal effects in pharmacological studies. Specifically, the authors did not test a control group in which a placebo was administered in between the two trials. In the absence of this crucial control, the data from this experiment cannot be taken as indication that the differences between pre-trials and post-trials are in fact caused by the drug.

The possibility of the current results being artifacts of repeated testing rather than effects of MDMA is further substantiated when one takes into account the temporal sequence of the two experiments reported in the publication. Three out of the four octopuses tested in the MDMA experiment summarized above (Experiment 2) were previously used as subjects in another test (Experiment 1), in which the same set-up was used but no drug was administered. In this test the authors report that all octopuses were presented with: (i) a female conspecific and a Chewbacca statue in the first trial, and; (ii) the same male conspecific as used in Experiment 2 and a Stormtrooper in the second trial. Thus, according to how the procedure is reported in the study, the three octopuses in question (i.e., subjects 1, 4, and 7) would have received three trials (trials 1 and 2 of Experiment 1, and pre-trial of Experiment 2) on one day, and the post-trial of Experiment 2 on the following day (Figure 1; Table S4, Edsinger and Dölen, 2018). If this was the case, then they were exposed to a novel female octopus in the morning, and then to the same male octopus across three trials (two trials in the afternoon of the same day, and one trial on the next day). As we will discuss later however, this seems to not actually be correct: these three octopuses in reality seem to have received only two trials (trials 1 and 2 of Experiment 1) before the post-trial of Experiment 2. Nonetheless, what this means is that these three octopuses were exposed to a novel female conspecific in the morning, another novel male octopus in the afternoon of the same day, and the same male conspecific on the next day. With this in mind, and because these three subjects account for a substantial fraction of the sample (75%), the overall pattern of data can be interpreted in alternative ways. For instance, a progressive extinction of the social response (perhaps due to habituation) could account for: (i) the reduction in the time spent with the social stimulus across the first trials, and; (ii) the subsequent increased response (dishabituation) in the post-trial of Experiment 2, after a 24 h delay (Figure 1). Supporting this view, previous independent studies showed that octopuses adjust their social response (e.g., by increasing avoidance behaviors) following repeated interactions with conspecifics (Cigliano, 1993; Tricarico et al., 2011). Alternatively, the repeated exposure to conspecifics could have allowed octopuses to learn that the social stimuli did not represent an actual threat (because they were constantly restrained in plastic cages), thus triggering bolder interactions on the last trial (i.e., post-trial of Experiment 2). The latter alternative interpretation appears plausible when one takes into account the solitary lifestyle of octopuses (Scheel et al., 2016; Amodio et al., 2019) and their reported cannibalistic attitude (Ibáñez and Keyl, 2010).

**Figure 1:**
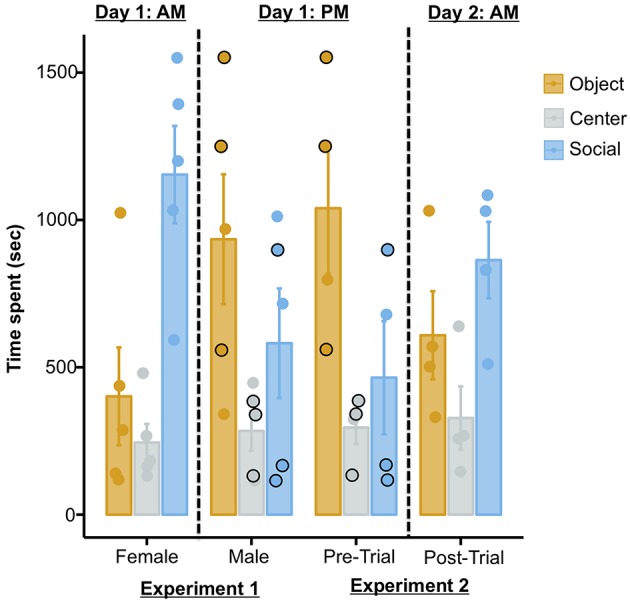
Plot showing the time spent in each chamber in Experiment 1 (left) and in Experiment 2 (right). The temporal sequence in which the two experiments were conducted is relevant only to octopuses that were tested in both experiments (i.e., 3/5 of the sample of Experiment 1 and 3/4 of the sample of Experiment 2, as reported in the original publication). The black outlined dots in the plot refer to the identical performances of three subjects in the second trial of Experiment 1 and in the pre-trial of Experiment 2 (see main text for detail). The plot was produced from raw data provided in the original paper (http://dx.doi.org/10.17632/z9t3x4p5kk.1#folder-73b7a7ef-1c00-49f3-addb-1756b279d653).

In addition to these problems with the experimental design, an unusual issue can be detected in the published raw data. Identical performances (i.e., time spent in each chamber) are reported for all octopuses that participated in both experiments across two independent trials (i.e., subjects 1, 4, and 7; Figure 1). The possibility that multiple octopuses spent the same amount of seconds in each of the three chambers on two consecutive test trials is theoretically possible, yet extremely unlikely. When enquiring about this issue with the authors of the original study, in a personal correspondence it was confirmed that for these three octopuses, the data from the second trial of Experiment 1 were also used for the baseline trial in the analysis of Experiment 2. Critically, this is not stated in the publication and raw data file, and not taken into account in the analyses; in all of these instances the data are reported and treated as independent.

Finally, data supporting the qualitative effect of MDMA on social behavior hinges exclusively on the observation that “after MDMA treatment, social interactions were characterized by extensive ventral surface contact” (Edsinger and Dölen, 2018), p. 3139). Yet, no additional details are provided, so that it is not known whether “extensive ventral surface contact” were performed exclusively/significantly more often in post-trials. However, even if either situation was the case, the physical exploration of a stimulus with multiple arms and the ventral surface of the body is not a *social-specific* response in octopuses. Octopuses frequently exhibit this behavior toward inanimate objects during foraging, play or problem solving (Fiorito et al., 1990; Kuba et al., 2003; Hanlon and Messenger, 2018). Hence, it is unclear whether these observations could be taken as strong qualitative evidence demonstrating the *prosocial* effect of MDMA in octopus. Similar studies with other model animals focused on actual social behaviors (e.g., adjacent lying in rodents, first-bite latency in fishes; (Kamilar-Britt and Bedi, 2015).

Taken together, we are skeptical about the claim that Edsinger and Dolen's experiments “provide the first functional evidence that the prosocial effects of MDMA are evolutionarily conserved in *O. bimaculoides*” (Edsinger and Dölen, 2018), p. 3139). We hope our arguments against this claim will foster a productive debate within the scientific community, thereby favoring the adoption of more solid experimental designs to test the effect of MDMA on octopus behavior, and providing the public with a critical tool to evaluate this innovative and highly featured study.

## Author Contributions

PA wrote the paper with critical additions and revisions by LO. NC and GF provided useful comments during revisions of the manuscript.

### Conflict of Interest Statement

The authors declare that the research was conducted in the absence of any commercial or financial relationships that could be construed as a potential conflict of interest.
